# Zoonotic Cutaneous Leishmaniasis, Afghanistan

**DOI:** 10.3201/eid1210.060076

**Published:** 2006-10

**Authors:** Michael K. Faulde, Gerhard Heyl, Mohammed L. Amirih

**Affiliations:** * Central Institute of the Bundeswehr Medical Service, Koblenz, Germany;; † Bundeswehr Medical Office, Munich, Germany;; ‡ Balkh Province Leishmaniasis Center, Mazar-e Sharif, Afghanistan

**Keywords:** Zoonotic cutaneous leishmaniasis, Mazar-e Sharif, outbreak, Rhombomys opimus, Phlebotomus papatasi, letter

**To the Editor:** Recent reports from Afghanistan have focused on the current status of war- and refugee-related anthroponotic cutaneous leishmaniasis (ACL), caused by *Leishmania tropica*, in Kabul City, refugee camps, and Fayzabad City (Badakhshan Province) (*1**–**4*). In central Asia, ACL is transmitted mainly by the sandfly *Phlebotomus sergenti* in urban or periurban environments (*5*).

Zoonotic cutaneous leishmaniasis (ZCL), caused by *L. major*, occurs autochthonously in Afghanistan, but little is known about its regional and seasonal distribution or other disease characteristics (*6**,**7*). Recently, cases have been reported in troops deployed to the Mazar-e Sharif area: 19 cases among the British military contingent in 2004, 186 among Dutch troops in 2005 (overall attack rate 20.9%), and 14 among German troops in 2005 (*8*). This yet-undescribed *L. major* variant is highly aggressive, often disseminates and causes nodular lymphangitis, and is associated with delayed or poor response to treatment with sodium stibogluconate or miltefosine.

Regional epidemiologic reports, when available from Afghan or international health authorities, usually mention cutaneous leishmaniasis cases without further elaboration. To evaluate the current threat and specific epidemiology of cutaneous leishmaniasis in Mazar-e Sharif, medical entomologic and epidemiologic field investigations were conducted in June and October 2005. Results show that ZCL, ACL, and visceral leishmaniasis (VL) are endemic to Mazar-e Sharif. Data from patients seeking treatment from March 21, 2004, through March 20, 2005, at the Balkh Province Leishmaniasis Center, Mazar-e Sharif, showed that of 3,958 cases, 3,782 (95.5%) were ZCL, 174 (4.4%) were ACL, and 2 (0.05%) were VL; the number of unreported cases during this time is unknown. A sharp increase (4.4- to 5.4-fold) in ZCL cases was found when data from July 21 through August 20 and from August 21 through September 20, 2004, (30 and 169 cases, respectively) were compared with data for the same periods in 2005 (163 and 744 cases, respectively). ZCL chiefly affects farmers, nomads, and refugees. By sex, cases occurred in 1,991 (52.6%) male and 1,791 (47.4%) female patients. By age group, the rate for ZCL in young children was similar to that for other age groups: 1,167 (30.9%) cases in children <4 years of age, 1,218 (32.2%) cases in children 5–14 years of age, and 1,397 (36.9%) cases in persons >15 years of age. In the leishmaniasis center, cutaneous infections are confirmed by seeing *Leishmania* parasites in Giemsa-stained smears obtained directly from skin lesions. Although sporadically confirmed by culture and PCR, ZCL and ACL are usually differentiated by specific clinical aspects, especially of skin lesions: dry lesions characterize urban ACL; wet lesions, often superinfected and disseminating, characterize rural ZCL.

In terms of seasonal patterns in Mazar-e Sharif, ZCL peaks in mid-October and most ACL cases occur during mid-February (Figure). Observations at the leishmaniasis center in Mazar-e Sharif indicate that the incubation period for ACL is 4–6 weeks (associated with the cold, wet season) and that for ZCL is 8–12 weeks (associated with the hot, dry season). The minimum incubation period of ZCL in German and Dutch patients was 7 weeks, a figure derived from the known period of troop deployment rather than from hospital records.

**Figure:**
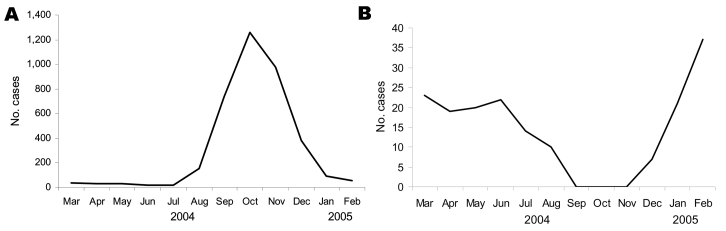
Seasonal distribution pattern of zoonotic cutaneous leishmaniasis (panel A) and anthroponotic cutaneous leishmaniasis (panel B) cases registered in the Balkh Province Leishmaniasis Center, Mazar-e Sharif, Afghanistan, March 21, 2004, through March 20, 2005.

The Mazar-e Sharif area, especially near the airfield, is semidesert, although extensive farming of seasonal crops is possible because of irrigation systems built in the 1980s. Irrigation canals are usually several kilometers long and 1.5–2 m deep and wide. Earth removed during canal construction is deposited as embankments that attract large numbers of the great gerbil, *Rhombomys opimus*, the main animal reservoir of *L. major* (*6*). Our studies showed an average of 3,380 *R. opimus* burrows per hectare, the highest density yet recorded, far exceeding the previous record of >1,000 rodent burrows per hectare in nearby Turkmenistan (*9*). Much lower *R. opimus* population densities were found at the Sakhi refugee camp, in the suburbs of Mazar-e Sharif, where Sherman box trapping for 18 nights was unsuccessful. Gerbil species were identified by photographs of live gerbils and by capture of 2. Both captured animals had positive results for *L. major* by microscopy and PCR. Because human enhancement of *R. opimus* habitat favors high infestations, the Mazar-e Sharif outbreak is an example of an anthropogenically induced emerging zoonosis. Sandfly surveillance was conducted in October 2005 by using unbaited CDC light traps (John Hock Co., Gainesville, FL, USA) and sticky traps placed in areas that were heavily infested with *R. opimus*. *Phlebotomus papatasi*, the principal vector of ZCL (*7*), was caught at low densities only, because of the cold night temperatures in October.

Our data suggest that foci of high-density enzootic ZCL occur in northern Afghanistan, especially in the Mazar-e Sharif area. ACL may also pose future problems because of its epidemic urban transmission potential (*10*). Effective disease surveillance and preventive measures should be promptly implemented to mitigate these health threats.
